# Ischemic Heart Disease Pathophysiology Paradigms Overview: From Plaque Activation to Microvascular Dysfunction

**DOI:** 10.3390/ijms21218118

**Published:** 2020-10-30

**Authors:** Paolo Severino, Andrea D’Amato, Mariateresa Pucci, Fabio Infusino, Francesco Adamo, Lucia Ilaria Birtolo, Lucrezia Netti, Giulio Montefusco, Cristina Chimenti, Carlo Lavalle, Viviana Maestrini, Massimo Mancone, William M. Chilian, Francesco Fedele

**Affiliations:** 1 Department of Clinical, Internal, Anesthesiology and Cardiovascular Sciences, Sapienza University of Rome, Viale del Policlinico, 155, 00161 Rome, Italy; paolo.severino@uniroma1.it (P.S.); damatoandrea92@gmail.com (A.D.); puccimariateresa@gmail.com (M.P.); fabio.infu@gmail.com (F.I.); francesco.adamo@uniroma1.it (F.A.); ilariabirtolo@gmail.com (L.I.B.); lucrezia.netti@gmail.com (L.N.); giulio.montefusco@gmail.com (G.M.); cristina.chimenti@uniroma1.it (C.C.); carlo.lavalle@uniroma1.it (C.L.); viviana.maestrini@uniroma1.it (V.M.); massimo.mancone@uniroma1.it (M.M.); 2 Department of Integrative Medical Sciences, Northeast Ohio Medical University, Rootstown, OH 44272, USA; wchilian@neomed.edu

**Keywords:** ischemic heart disease, microcirculation, atherosclerosis, coronary blood flow, myocardial infarction, ion channels

## Abstract

Ischemic heart disease still represents a large burden on individuals and health care resources worldwide. By conventions, it is equated with atherosclerotic plaque due to flow-limiting obstruction in large–medium sized coronary arteries. However, clinical, angiographic and autoptic findings suggest a multifaceted pathophysiology for ischemic heart disease and just some cases are caused by severe or complicated atherosclerotic plaques. Currently there is no well-defined assessment of ischemic heart disease pathophysiology that satisfies all the observations and sometimes the underlying mechanism to everyday ischemic heart disease ward cases is misleading. In order to better examine this complicated disease and to provide future perspectives, it is important to know and analyze the pathophysiological mechanisms that underline it, because ischemic heart disease is not always determined by atherosclerotic plaque complication. Therefore, in order to have a more complete comprehension of ischemic heart disease we propose an overview of the available pathophysiological paradigms, from plaque activation to microvascular dysfunction.

## 1. Introduction

Although over the last years clinical practice strategies have evolved optimizing prevention and treatment for ischemic heart disease (IHD), the consequences of this condition represent a significant burden on human health, in terms of mortality and morbidity [1]. Currently, basic, translational, and clinical data have provided a massive amount of information about the etiology of myocardial ischemia. However, both clinical, angiographic and autoptic findings suggest a complex pathophysiology of IHD [2,3,4,5,6,7,8], which goes beyond the conventional and simplistic role of atherosclerosis. For this reason, it is necessary to overcome the concept that IHD is always an atherosclerotic disease synonym. It is important to analyze the proposed paradigms of IHD in order to better examine this complicated disease and to provide future perspectives. In the literature, there is a copious number of reviews on this topic [9,10,11]. If the readers were wondering why we are going to propose another review and another paradigm, our response would be that currently there is no well-defined evaluation of IHD pathophysiology satisfying all the pathophysiological observations. Simply, the heart may be equated to an airplane where more than one redundant regulatory mechanism has to fail jointly to create an event. IHD pathophysiology is more complex and multifaceted than a single, simplistic, cause–effect event.

## 2. Coronary Macrocirculation Involvement in the Ischemic Heart Disease Pathophysiology

In the coronary tree, the proximal section is represented by epicardial coronary arteries, with diameters ranging from 250 μm to 2–5 mm [12]. These vessels have a capacitance function and offer merely a tiny contribution to coronary vascular resistance under normal conditions. Epicardial arteries are responsive to flow dependent dilatation and are subjected to shear stress that vary every heartbeat, during the phasicity of coronary blood flow. The vasodilatory effects of shear stress are mediated by endothelial-dependent vasodilatation.

### 2.1. Ischemic Heart Disease and the Hydraulic Paradigm: Role of Coronary Artery Disease and Vasospasm

**Figure 1:**
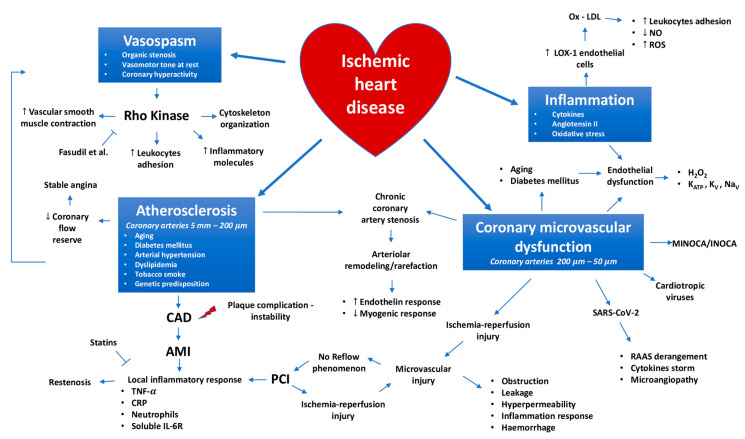
Schematic representation of pathophysiological mechanisms involved in ischemic heart disease. Ischemic heart disease is determined by an imbalance of the cross talk between myocardial energy state and coronary blood flow. This is due to several conditions. In particular, atherosclerosis, coronary microvascular dysfunction, inflammation and vasospasm contribute to the multifaceted and complex pathophysiology of ischemic heart disease. CAD: coronary artery disease; AMI: acute myocardial infarction; PCI: percutaneous coronary intervention; TNFα: tumor necrosis factor alpha; CRP: C-reactive protein; IL-6R: interleukin-6 receptor; SARS-CoV-2: severe acute respiratory syndrome coronavirus 2; RAAS: renin–angiotensin–aldosterone system; MINOCA: myocardial infarction with non-obstructive coronary arteries; INOCA: ischemia with non-obstructive coronary arteries; H_2_O_2_: hydrogen peroxide; K_ATP_: ATP-sensitive potassium channel; K_v_: voltage-gated potassium channel; Na_v_: voltage-gated sodium channel; LOX-1: oxidized low-density lipoprotein receptor 1; Ox-LDL: oxidized low-density lipoprotein; ROS: reactive oxygen species; NO: nitric oxide; ↑: increase; ↓: decrease.

According to the conventional IHD pathophysiological point of view, an obstructive plaque that inhibits blood flow, through the coronary artery, triggers myocardial ischemia. Coronary artery disease (CAD) is defined by the presence of an obstructive atherosclerotic plaque, which causes a blood flow reduction to the myocardium (Figure 1). This “culprit” stenosis represents the final stage of the complex atherosclerotic process. The latter is mostly associated with contemporary lifestyles. However, atherosclerosis was common also in preindustrial populations [13]. Only in the recent past, it was coined the concept of “critical” coronary stenosis, from a “hydraulic” point of view. At rest, coronary blood flow is maintained up to the point of development of a critical stenosis by the process of autoregulation, defined as the capacity to preserve blood flow during changes in perfusion pressure, with constant metabolic needs. Although the exact mechanisms underlying autoregulation have not been unequivocally proven, myogenic and metabolic mechanisms play a key role. When an atherosclerotic plaque obstructs over 70% of the luminal cross-sectional area with 50% coronary diameter reduction, it increases the proximal resistance significantly and decreases distal coronary perfusion pressure. In this situation, autoregulation is able to maintain basal coronary blood flow, but the dilator reserve is compromised. This may lead to a non-symptomatic condition at rest, but insufficient flow at high metabolic demands, for example, during physical exercise [14].

“Hydraulic” modification of coronary arteries has been observed with aging [15,16]. Aging represents a physiological mechanism. It is an independent cardiovascular risk factor, which may have a great impact on IHD pathogenesis and pathophysiology, because it could act with diabetes, arterial hypertension, dyslipidemia and tobacco smoke. Aging determines several changes in both coronary epicardial arteries and microvasculature. Its pathogenetic role is strongly influenced by genetic predisposition and environmental features. It is responsible for endothelial layer integrity loss, arterial stiffness, loss of vessel’s elasticity and reduced vascular adaptability to physical forces related to coronary blood flow [15,16]. These aspects facilitate CAD progression and complications. From the histopathological point of view, aging is associated with vascular fibrosis, increase in collagen deposition and elastin reduction, intimal thickening, subendothelial cholesterol and phospholipid storage. Acting together with cardiovascular risk factors, aging determines the progression of atherosclerotic lesions and arterial stiffness resulting in impaired myocardial perfusion. Moreover, aging promotes an increased expression of cyclooxygenase (COX) 1 and 2, thromboxane A, von Willebrand factor and factor VIII promoting platelet aggregation and a hypercoagulative state. Also, inflammation response is enhanced with aging and participates in “hydraulic” modification of coronary arteries, facilitating arterial stiffness and atherosclerotic plaque growth. In this case, macrophages are the main leukocytes sub-type involved in the vascular inflammation damage induced by aging [15,16] (Figure 1).

Several years ago, it was proposed that myocardial ischemia might be caused by coronary arteries spasms: vasospasm of one of the main coronary arteries are able to cause complete occlusion. In 1959, Prinzmetal described a syndrome of angina with electrocardiographic ST-segment elevation, in absence of coronary obstruction [17]. “Prinzmetal” angina is caused by transient vasospasm with an acute reduction of coronary blood supply, rather than by a myocardial metabolic demand increase [18,19]. The significance of coronary spasm has been demonstrated in other clinical scenarios with an involvement of the coronary microcirculation. Moreover, it is now recognized that spasm can be initiated by several factors, i.e., vasomotor tone at rest, segmental epicardial coronary hyperactivity, and an organic stenosis (Figure 1). Coronary spasm frequently appears also at sites of hemodynamically significant atherosclerotic stenosis, both in acute and chronic settings. Spasm of atherosclerotic lesions may provoke myocardial ischemia, with coronary vasomotor response related to the plaque burden [20]. Moreover, in patients with focal vasospasm, angiography demonstrates non significant atherosclerosis [21], proposing that spasm may occur both during early and advanced stages of disease [22]. A downstream vasospastic effector is Rho-associated protein kinase (ROCK), a small ubiquitously expressed G protein involved in several cellular functions, such as circulating leukocytes adhesion, smooth muscle contraction, and actin cytoskeleton organization [23,24] (Figure 1). In addition, ROCK increases the expression of inflammation related molecules, thrombosis and fibrosis [25]. Moreover, ROCK is involved in the pathophysiology of angina pectoris, coronary vasospasm, hypertension, and pulmonary hypertension. For these reasons, ROCK activity can be a useful novel biomarker for the assessment of disease severity and therapeutic responses in cardiovascular medicine. Beyond Fasudil, other ROCK inhibitors are currently under development for many indications [23,24]. Fasudil, through molecular pathway PI3K/Akt/eNOS stimulation, may increase myocardial nitric oxide (NO) concentration, restoring the related ischemic post conditioning cardioprotection, impaired by hypercholesterolemia [26,27] (Figure 1).

### 2.2. The Biological Paradigm: The Bidirectional Link between Inflammation and Myocardial Ischemia

In the last decades, some authors suggested that the course of atherosclerosis is supported by inflammation, from its beginning to thrombotic complications. In fact, it has been hypothesized that inflammation stimulates atherosclerosis initiation and evolution. It also contributes to the acute thrombotic complications of atherosclerotic plaque [28,29,30,31,32,33]. However, inflammation represents a response to myocardial ischemia, which involves myocardium initially becoming systemic later.

#### 2.2.1. Inflammation as Cause of Ischemic Heart Disease

In physiological conditions, leukocytes are not activated by endothelium. However, inflammation changes the interaction between the endothelium and leukocytes severely, leading to endothelial expression of adhesion molecules, that bind to leukocytes, persevering and enhancing a local inflammatory response. Moreover, local inflammation produces some proteolytic enzymes making the atherosclerotic cap prone to rupture.

Adaptive immune response plays an important role in atherosclerotic lesion development and its clinical manifestations [34]. Moreover, they seem to be involved in the pathogenesis of traditional cardiovascular risk factors, as arterial hypertension, diabetes mellitus and metabolic syndrome [35,36], and seem to be deregulated on a genetic basis (i.e., single nucleotide polymorphisms (SNPs) for interleukin (IL)-6 receptor) [37]. Some clinical studies hypothesized the correlation of inflammation circulating markers, as C-reactive protein (CRP) and homocysteine [38,39], with the susceptibility to develop IHD and with an associated worse prognosis.

IHD prognosis may be improved by antibiotic treatment [40,41] using the rationale that reducing the amount of bacterial endotoxin, secondary to an infection, would minimize inflammation. Moreover, other emerging non-traditional risk factors, such as elevated plasma and tissue levels of oxidized low-density lipoprotein (oxLDL), seem to be equally important [42,43]. OxLDL are considered to play a fundamental role in the entire process of atherogenesis, from plaque formation to plaque destabilization. OxLDL determine endothelial dysfunction, stimulate generation of reactive oxygen species (ROS), inhibit NO synthesis, and enhance monocyte adhesion to activated endothelial cells [44,45]. In addition, oxLDL can induce vascular smooth muscle cells (VSMCs) migration and proliferation, and is avidly ingested by macrophages, resulting in foam cell formation. OxLDL may also induce apoptosis and necrosis of vascular endothelial cells, VSMCs and macrophages [17,19,26,28]. In this regard, oxLDL increased levels relate to plaque instability with a significant positive correlation with myocardial ischemia severity in human coronary atherosclerotic lesions. Moreover, the more severe lesions contain a significantly higher percentage of oxLDL–positive macrophages [46]. These events are modulated by the overexpression of the lectin-like oxidized LDL (LOX-1), a scavenger receptor that selectively uptakes oxLDL into endothelial cells. LOX-1 is highly modulated by stimuli such as cytokines, mechanical forces, angiotensin II, oxidative stress and directly by the occurrence of oxLDL [47,48,49]. At the final stages of atherogenesis, oxLDL contributes to the development of endothelial cells apoptotic death, possibly via overexpression of LOX-1. The latter seems to be mediated by the overproduction of ROS (Figure 1). In particular, the modulation of LOX-1 by oxLDL leads to the superoxide anions overproduction. In presence of NO, they promote the peroxy-nitrite (ONOO^−^) generation, a highly reactive endothelial cells toxic oxidant that leads to cell apoptosis [47]. In atherogenesis and IHD, the pathophysiological role of cholesterol and its relationship with inflammation has been widely described and it may counteract with several approaches. For example, the use of statins and ezetimibe is associated with reduction of LDL blood values and cardiovascular diseases related to hypercholesterolemia. Moreover, statins reduce also the inflammation related to atherosclerosis. Novel targets to reduce hypercholesterolemia have recently been described [50,51]. The adenosine triphosphate (ATP) citrate lyase is upstream of hydroxymethylglutaryl-CoA reductase (HMG-CoA reductase), the enzyme inhibited by statins. It is involved in cholesterol biosynthesis and inhibited by bempedoic acid. Bempedoic acid has demonstrated to reduce LDL cholesterol when associated with statins [50]. Proprotein convertase subtilisin/kexin type 9 (PCSK-9) is responsible for LDL receptor degradation and inhibits its migration to cells’ membranes. For this reason, PCSK-9 inhibition reduces circulating LDL cholesterol and its related major cardiovascular events [51].

Diabetes mellitus promotes the quick progression and complications of atherosclerotic disease [52]. It enhances the vascular damage induced by other cardiovascular risk factors, such as dyslipidemia. Hyperglycemia enhances ROS production that promote ox-LDL formation. Diabetes mellitus is also associated with more frequent plaques rupture and asymptomatic myocardial ischemia [52]. Plaque inflammation and their necrotic area are more widespread in patients affected by both type 1 and 2 diabetes mellitus. Moreover, inflammatory infiltrates are mainly constituted by T-cells and macrophages. In particular, hyperglycemia and hemoglobin A1C (HbA1c) levels are associated with inflammation entity and atherosclerotic plaques core size and calcification. In diabetes mellitus, coronary calcification is driven by ROS, which stimulate several mechanisms, such as vascular inflammation induced by nuclear factor kappa-light-chain-enhancer of activated B cells (NF-κB), protein kinase C (PKC) pathway and formation of advanced end glycation products (AGEs) (Figure 1). These mechanisms promote the expression of an osteoblast-like phenotype for VSMCs, which synthetize several proteins normally involved in osteochondrogenesis [52]. Moreover, chronic kidney disease often affects patients with diabetes mellitus and cardiovascular disease. It is characterized by hyperphosphatemia that promotes the osteogenic related gene expression in VSMCs and facilitates vascular calcium deposition. For these reasons hyperphosphatemia closely relates with plaque calcification extension [52].

Inflammation has been proposed as a cause of plaque vulnerability and potential rupture, responsible for acute atherothrombotic vascular occlusion and tissue infarction. In fact, in patients presenting acute coronary syndrome (ACS), angiographic studies have identified culprit lesions that do not cause marked stenosis. It is now evident that the plaque activation rather than stenosis, precipitates ischemia and infarction. Inflammation plays a key role in CAD and other atherosclerotic manifestations [29,53,54,55]. Immune cells dominate early atherosclerotic lesions. Moreover, their effector molecules accelerate lesions progression, and inflammation activation can elicit ACS. So, what causes a silent atherosclerotic lesion to rupture finally? Activated macrophages, T cells, and mast cells at sites of plaque rupture produce several types of molecules—inflammatory cytokines, proteases, coagulation factors, radicals, and vasoactive molecules—that can destabilize lesions [56,57,58,59]. They inhibit the formation of stable fibrous caps, attack collagen in the cap, and initiate thrombus formation. All these reactions can conceivably induce the activation and rupture of plaque, thrombosis, and ischemia. The balance between inflammatory and anti-inflammatory activity controls the atherosclerosis progression. Metabolic factors may affect this process in several ways. Obviously, they contribute to artery lipid deposition, initiating new rounds of immune-cell recruitment. Furthermore, the adipose tissue of patients with metabolic syndrome and obesity produces inflammatory cytokines, particularly tumor necrosis factor (TNF) and interleukin-6 (IL-6) [33].

#### 2.2.2. Inflammation as Consequence of Ischemic Heart Disease

Acute myocardial infarction (AMI) produces a significant local inflammatory response, which starts in the myocardium and propagates systemically through the blood stream. Lots of inflammatory cytokines, such as tumor necrosis factor alpha (TNFα) and various chemokines that are weakly represented in healthy hearts, reach high levels during myocardial infarction [60,61,62]. Elevation of acute phase reactants such as CRP and increased peripheral white blood cell count, especially neutrophils, are common during ACS [63,64]. Several studies have reported correlations between increased neutrophil count in peripheral blood and short-term post ST-elevation myocardial infarction (STEMI) adverse outcomes, including mortality [65,66,67]. The panel of inflammation related molecules produced during IHD is growing: high levels of soluble interleukin-6 receptor (sIL-6R) can predict future cardiovascular events and mortality in STEMI patients [68]. In the biological atherothrombosis associated with IL-6 pro-inflammatory pathway, interleukin-1β (IL-1β) stimulates proliferation and hypertrophy of VSMCs. It has a procoagulant effect and facilitates the leukocytes recruitment to the vessels’ walls [69]. For this reason, a human monoclonal antibody directed versus IL-1β called Canakinumab reduces inflammatory response, atherothrombosis and cardiovascular events [69]. It diminishes plasmatic CRP and IL-6 in patients with myocardial infarction history, without influencing cholesterol levels [69]. Moreover, decreased serum vasostatin-2 level is associated with post-ischemic chronic heart failure and with major cardiovascular events [70]. Galectin-3 binding protein plasma levels are associated with long-term mortality in CAD, independent from plaque morphology [71]. In this context, statin treatment seems to mitigate the cellular inflammatory response after the myocardial infarction, reducing leukocyte and neutrophil cell counts [72]. Also percutaneous coronary intervention (PCI) may induce a local inflammatory response that contributes to restenosis, together with other factors, such as the possible interaction between stent materials and passive red blood cell membrane electrical parameters [73] (Figure 1). Inflammation originates from several molecular signals. Injured cardiomyocytes start innate immune responses involving neutrophils, ROS, toll-like receptors, myeloperoxidase, interleukins [73,74,75].

A crucial scenario is the myocardial ischemia-reperfusion injury. In fact, the process of myocardial reperfusion can induce cardiomyocyte death paradoxically. Post-ischemic intracellular edema represents the inflammatory response to the acute ischemic insult and so, it can expose the myocardium to adverse effects related to ROS, during ischemia-reperfusion [76]. In addition, free fatty acids strongly increase during reperfusion and their toxic effects on cellular membranes lead to arrhythmias and cardiac function decrease [77,78].

Thrombus formation in correspondence to an unstable coronary plaque may associate with low-grade endotoxemia [79]. Higher levels of *Escherichia Coli* derived endotoxin lipopolysaccharide (LPS), and other inflammatory products as CPR, tissue factor, and several cytokines, are present in patients with STEMI, compared to control group and to stable angina patients. *Escherichia Coli* derived LPS levels positively correlate with the levels of Zonulin and P-selectin, suggesting a role of intestinal microbiota in coronary thrombosis after its translocation in systemic circulation [79]. However, the link between *Escherichia Coli* derived LPS and STEMI is not univocal because the higher levels of LPS in STEMI patients may represent a consequence of infarction related inflammation, more than the cause of myocardial infarction. According to the complexity of IHD pathophysiology, LPS and inflammation may destabilize atherosclerotic plaques, contributing to their evolution, together with other factors, rather than being a primary cause of IHD.

### 2.3. Coronary Circulation Involvement during HIV Infection and Antiretroviral Therapy

Human immunodeficiency virus (HIV) infection is associated with an increased risk of AMI, ischemic stroke and heart failure [80,81,82,83,84,85]. The underneath mechanism driving cardiovascular disease (CVD) risk associated with HIV is not clear, but likely involves a combination of factors including the virus itself, the side effects of antiretroviral therapy, the burden of coronary heart disease traditional risk factors and non-traditional risk factors (i.e., hepatitis C, substance use or abuse) [86]. HIV-infected patients could show a peculiar scenario. Virtual Histology-Intravascular ultrasound (IVUS) analysis showed a high prevalence of unstable plaque morphology rich in necrotic tissue. HIV-related plaque seems to be different from those of the general population: less calcific and more necrotic, with a thick-cap. This suggests a peculiar pathophysiological mechanism for HIV-related atherosclerosis that is independent from traditional risk factors [87]. In fact, HIV infection itself seems to play a crucial role in the development of atherosclerosis: coronary plaque is positively associated with immune activation marker CD8^+^CD38^+^DR and E-selectin, a marker of endothelial inflammation [88]. HIV also plays a pathogenic effect on cholesterol metabolism, through down-modulation and functional impairment of ATP-binding cassette A1 (ABCA1) cholesterol transporter through the HIV-1 protein negative factor (Nef) [89]. In non HIV-infected patients and animal models, CD4^+^ cells modulate inflammation and consequently atherosclerosis. During HIV infection, this equilibrium is imbalanced and the inflammatory process together with opportunistic infections lead to an increased cytokine expression and vascular damage [90,91,92]. Additionally, antiretroviral (ART) therapy is an important risk factor for CVD, in HIV patients [93]: in fact, despite the ART therapy, immune activation persists in people with HIV infection, contributing to accelerated atherosclerosis and promoting coronary atherosclerotic plaques rupture [94]. Moreover, ART therapy worsens the circulating lipid profile. Several biological mechanisms link HIV infection to myocardial ischemia. Among HIV proteins, glycoprotein 120 (gp120) enhances endothelin-1 release from endothelium, while Nef and transactivator of transcription (Tat) contribute to endothelial dysfunction and inflammation [95,96,97]. Plasma circulating virus and immune dysregulation are associated with CVD. In particular, the presence of atherosclerosis and higher cardiovascular mortality has been described in patients with reduced CD4^+^ T lymphocytes and detectable viremia [95,98,99]. T cells activation contributes to endothelial dysfunction, while higher CD163 plasma levels, determined by increased macrophages activation, relates to arterial inflammation [95,100]. During HIV infection, the presence of circulating CD14 represents a microbiota translocation marker. It associates with higher circulating levels of TNF and IL-6 and it may have a role in the faster evolution of the atherosclerotic process [95]. According to a better comprehension of cardiovascular involvement during HIV infection, a differentiation of patients based on circulating biomarkers may be useful also from a clinical point of view. Increased N­terminal pro­ B­type natriuretic peptide (NT-proBNP), ST-2 and growth-differentiation factor 15 levels define the cardiac phenotype, which seems to develop more frequently pulmonary arterial hypertension and right ventricle dysfunction. Instead, higher d-dimer, IL-6 and CRP characterize the inflammatory phenotype, which develops mainly microvascular and diastolic dysfunction [94,101]. In treated HIV patients, increased levels of plasmatic PCSK-9, a marker of lipid metabolism, are associated with coronary endothelial dysfunction. It is confirmed by the presence of higher levels of endothelial damage markers, such as thrombomodulin and P-selectin [102]. HIV patients who are co-infected with other viral agents, such as cytomegalovirus (CMV), together with a long treatment history of ART therapy administration, have a higher number of circulating CD8^+^ T lymphocytes. They show a quicker atherosclerosis progression [95,103].

## 3. Role of Coronary Microcirculation in the Pathophysiology of Ischemic Heart Disease

In the coronary tree, arterioles with 50–200 μm in diameter represent the microcirculation that reflects approximately 60% of coronary resistance [12].

In the last decades, coronary microcirculation function and structure abnormalities have been described as a relevant IHD pathogenic mechanism. Coronary microvascular dysfunction (CMD) represents a common pathophysiological mechanism of type II myocardial infarction [104]. CMD determines an increase in flow resistance, leading to myocardial ischemia, in response to reduced perfusion pressure [105]. The term ‘microvascular angina’ for this patient population was used for the first time in 1985, referring to ischemia triggered by an altered vasoregulation of the coronary microcirculation. Subsequently, several studies lead to a better, but not full, knowledge of CMD. From a clinical point of view, the terms ‘coronary microvascular dysfunction’ and ‘microvascular angina’ seem to be more appropriate, rather than ‘angina with normal coronary arteries’ [106]. A pre-existing transient or permanent microvascular dysfunction may contribute to the development and prognosis of ACS, via reduction of coronary blood flow. Moreover, a shear stress alteration aggravates endothelial dysfunction and enhances thrombus formation, in epicardial arteries [107]. A chronic coronary artery stenosis brings arteriolar inward remodeling and rarefaction, as well as blunting of myogenic responses and increased vasoconstrictor responses to endothelin, due to a loss of endothelin B receptor-mediated vasodilation [108,109,110]. Nevertheless, as seen in severe hypertrophy, a two-fold increase in left ventricular mass may drastically reduce coronary flow reserve in a non-stenotic artery, despite the absence of mean aortic pressure change [111]. In addition, CMD may associate with intramural vessels structural abnormalities, due to hypertrophy and hyperplasia of VSMCs [112], or small vessels infection, due to cardiotropic viruses [113].

In clinical practice, modern cardiologists consider cardiovascular imaging as an ineluctable tool to make the right diagnosis. However, in humans, coronary microcirculation is not visualized with routine invasive and non-invasive imaging tools. Several coronary blood flow measuring methods are used to evaluate systemic microvascular function, providing just indirect information about the microcirculation. Among these, there are the ankle-brachial index and toe-brachial index, for the assessment of microvascular peripheral artery disease [111,114], and Doppler echocardiography on coronary arteries origin, for coronary microvascular dysfunction assessment [111,115].

For this reason, the real role of coronary microcirculation in IHD pathophysiology is still not totally recognized and understood.

### 3.1. The Mechanistic Point of View of Coronary Microvascular Dysfunction Pathophysiology

In a substantial proportion of patients who underwent mechanical reperfusion by PCI, the opening of the obstructed epicardial vessel does not accomplish myocardial reperfusion. In this scenario, the increase of myocardial microvascular resistance and the reduction of myocardial perfusion may provide a pathophysiological link between microvascular obstruction and cardiac remodeling and death after AMI and PCI. Three mechanisms may be responsible for coronary microvascular obstruction: distal embolization, ischemia-reperfusion related injury and individual susceptibility [116]. Coronary embolism is frequent in several conditions associated with high risk of systemic embolization, such as chronic atrial fibrillation, infective endocarditis, prosthetic heart valves, cardiac tumor and PCI [117,118]. Microembolization should be detected by Doppler ultrasound, during PCI. In fact, coronary microemboli are observed in each of the PCI procedural phases [119,120]. Interestingly, systemic inflammatory response and microvascular impairment after PCI are significantly higher in patients with non ST segment elevation myocardial infarction (NSTEMI), than patients without NSTEMI [119,120]. Platelets adhere and aggregate to inflamed microvessel endothelium, where they release several molecules, such as thromboxane and ROS. After activation, platelets may support neutrophil extracellular traps (NETs) formation, because they express high mobility group box-1 (HMGB1). This mechanism may have a central role in microthrombosis and no reflow phenomenon [121,122]. Moreover, the interaction among platelets and endothelial cells through CD40L and its receptor, respectively, mediates local inflammation, predisposing to microvascular thrombosis [121]. During PCI, adjunctive therapies that may reduce microemboli formation include intensive antiplatelet therapy, coronary vasodilators and embolization devices protection. At the same time, the addition of ranolazine to standard anti-ischemic therapy seems to determine a significant improvement of ECG stress-test tolerance, improving the myocardial ischemic threshold. Moreover, ranolazine reduces exercise angina symptoms and ischemic related arrhythmias [123]. Ischemia-reperfusion damage is typically mediated by local microvascular responses: activated endothelial cells in all microcirculation segments produce more oxygen radicals and less NO, in the initial period following reperfusion. The resulting imbalance between superoxide and NO leads to the production and release of inflammatory mediators (e.g., platelet-activating factor, tumor necrosis factor) and enhances the adhesion molecules biosynthesis that mediates leukocyte–endothelial cell adhesion [124].

Another scenario is represented by myocardial ischemia in hypertrophic cardiomyopathy (HCM). HCM is the most common inherited cardiovascular disorder, affecting 1 in 500 individuals worldwide [125]. From the histopathological point of view, HCM is characterized by microvascular rarefaction, intimal hyperplasia, medial hypertrophy, increased collagen deposition and small intramural coronary arteries decreased luminal size. These morphological alterations lead to microvascular dysfunction, decreased coronary flow reserve and myocardial fibrosis [125,126,127,128,129]. The microvascular structural abnormalities are revealed by an inadequate increase in myocardial blood flow after intravenous administration of the vasodilator dipyridamole and on positron-emission tomography (PET), in the majority of patients with HCM [130,131]. Moreover, a weak vasodilator response in the endocardium is proportional to the extension of wall hypertrophy, during stress perfusion-cardiac magnetic resonance imaging (MRI) [129,132].

Arterial hypertension increases shear stress induced by blood flow on the endothelial layer. It worsens atherosclerotic lesions and endothelial dysfunction. Arterial hypertension determines increased contraction of VSMCs, which protects microcirculation from hypertension related damage and reduces the development of myocardial edema. Conversely, blood flow velocity and shear stress increase determine the imbalance of endothelial function. It represents an example of how abnormal mechanical forces are transduced in functional and molecular dysregulation [121,133]. In epicardial vessels, CMD seems to trigger debris, vasoconstrictor and pro-inflammatory substances release from culprit lesion. They enhance microvascular dysfunction, promoting the microvascular function worsening. Conversely, CMD may have consequences also on hemorheological features of blood flow in epicardial arteries, worsening CAD and establishing a vicious circle, which contributes to myocardial ischemia [134,135].

In patients with ACS who underwent PCI, coronary microcirculation should be inadequately perfused, despite the treatment of the culprit lesion. This condition is known as microvascular injury (MVI). It is related to ischemia, occurs in the infarction core zone and develops during reperfusion. At microcirculation, MVI represents the main expression of ischemia reperfusion damage and it is known as no reflow phenomenon or microvascular obstruction, from the clinical point of view. Due to no reflow phenomenon, the infarct area shows lesser healing and greater size. No reflow phenomenon contributes to eccentric hypertrophy, left ventricle remodeling and heart failure [136]. It is associated with the duration of coronary occlusion, the Thrombolysis In Myocardial Infarction (TIMI) score before the reperfusion, the hyperglycemic state and aging [137]. From the histopathological point of view, MVI is characterized by the presence of microvascular obstruction, leakage, and hyperpermeability until intra-myocardial hemorrhage, which is characterized by erythrocytes extravasation [138,139] (Figure 1). Microvascular leakage is a critical consequence of MVI because it involves a myocardial area greater than that one involved in acute ischemia. Moreover, the extension of microvascular leakage is associated with ventricular remodeling and dilatation severity [140].

Interstitial edema formation is the consequence of microvascular leakage and permeability. During ischemia, it begins early being sustained by the inflammation response and it enhances during reperfusion, due to hyperemic reaction and vasoactive molecules wash-out. Edema worsens CMD, during no reflow phenomenon [137]. Moreover, pericytes contraction may also contribute to microvascular constriction and dysfunction [136]. MVI is also associated with local thrombus formation, which worsens the no reflow condition. During PCI, the balloon expansion and stent application determine the distal massive migration of microemboli and thrombotic residues. These, together with several substances, such as endothelin, neuropeptide Y, cholesterol crystals, cytokines, thromboxane and serotonin, may promote microvascular vasoconstriction [136,141]. From the functional point of view, enhanced microvascular endothelium dependent vasoconstriction and impaired endothelial dependent vasodilation have been observed during MVI [142,143]. Despite the absence of residual angiographic epicardial stenosis, the ischemia reperfusion phenomenon is associated with the coronary blood flow reserve reduction. The coronary flow reserve reduction is a marker of CMD and it occurs independently by the revascularization of the main epicardial arteries [144]. During MVI, several mechanisms contribute to the vascular wall damage, such as inflammation, leukocytes action, complement pathways activation, mitochondrial dysfunction and ROS induced cell death. During ischemia reperfusion injury, ROS production is induced by the telomerase reverse transcriptase (TERT) genetic loss and it is associated with an imbalance of endothelial NO production. Nucleotide-binding domain oligomerization domain-like receptor containing pyrin domain 3 (NLRP3) contributes to the local cytokines production and cell death related inflammation [142]. The endothelial damage begins during ischemia with microvessel’s lumen enlargement. During reperfusion, it continues with thickening and destruction of endothelial cells. Moreover, the microvascular leakage and hyperpermeability are associated with a wide loss of cell junctions, after reperfusion [138]. Conversely the heat shock protein specifically expressed by endothelial cells during ischemia reperfusion, named HSPA12B, acts via an endothelial NO synthase (eNOS) mediated angiogenesis mechanism, attenuating the cardiac remodeling and reducing the no-reflow phenomenon. HSPA12B diminishes vascular leaking and endothelial damage, improving microvascular function [145]. Cardiac magnetic resonance represents the main technique to identify and assess MVI and no reflow phenomenon. Also, microvascular leakage may be identified with CMR and it has a negative prognostic meaning for left ventricular remodeling [137].

There are several treatments to deal with the ischemia reperfusion injury, guaranteeing cardioprotection. Pharmacological and physical treatments, as well as ischemic conditioning have been described [146]. Regarding physical treatment, electrical nerve stimulation and hypothermia have been proposed [146]. The rationale behind ischemic conditioning consists in the activation of several protective molecular pathways, through the induction of short periods of ischemia and reperfusion, before or after the main insult, as happens in the ischemic preconditioning and ischemic postconditioning respectively. Remote ischemic conditioning consists in the application of short periods of ischemia reperfusion injury in a different site from the heart [146]. In this regard, remote ischemic conditioning protects mitochondrial function and the production of NO and protein kinase G (PKG). Ischemic preconditioning counteracts the NO synthase uncoupling, reducing nitrogen species and ROS production.

Several pharmacological approaches against ischemia reperfusion injury have been studied [146,147,148,149,150,151,152]. During reperfusion, the use of exenatide, an incretin mimetic, or glucose-insulin-potassium, together with remote ischemic conditioning, is associated with infarct size reduction. The same effects were obtained through the use of recombinant human angiopoietin-like protein 4 (ANGPTL4), which contrasts the no reflow phenomenon and the intra-myocardial hemorrhage. The caspase 1 related pyroptosis inhibitor, named VX-765, as well as necrostatin-1 and Z-VAD administered together with P_2_Y_12_ inhibitors, contrast necroptosis and apoptosis, determining infarct size reduction and cardioprotection [146,148]. Regarding P_2_Y_12_ inhibitors, the “The Platelet Inhibition to Target Reperfusion Injury” (PITRI) trial is ongoing. It is evaluating if Cangrelor administration may contrast reperfusion injury [147]. During reperfusion, the simultaneous administration of two molecules acting on the same pathway, such as tetrahydrobiopterin and L-Arginine, promotes the NO production and reduces infarct area size [148]. During reperfusion, the intravenous administration of metoprolol reduces neutrophil-platelet aggregate formation while it reduces myocardial work and cardiomyocytes metabolic demand, during ischemia. When infused before reperfusion, metoprolol reduces infarct area size, as demonstrated by “Metoprolol in Cardioprotection During an Acute Myocardial Infarction” (METOCARD-CNIC) trial [149]. There are many ongoing trials to find an adequate treatment to induce cardioprotection, in patients with STEMI who undergo PCI. In this regard, the “Combined Application of Remote and Intra-Coronary Ischemic Conditioning in Acute Myocardial Infarction” (CARIOCA) trial evaluates the effect of ischemic post-conditioning and remote ischemic conditioning on the clinical outcome, before reperfusion [148]. The “COMBi-nAtion Therapy in Myocardial Infarction” (COMBAT-MI) trial evaluates the combined effect of remote ischemic conditioning and exenatide administration [148]. “N-acetylcysteine in Acute Myocardial Infarction” (NACIAM) trial reported a reduction of an extra 5.5% infarct area size in patients who received additional N-acetylcysteine, beyond nitroglycerine infusion [148,150]. The “Acute Myocardial Infarction with Hyperoxemic Therapy II” (AMIHOT-II) trial demonstrated the role of supersaturated oxygen, in the reduction of anterior infarct area extension [151]. The β adrenoceptors stimulation promotes NO production and mitochondrial K_ATP_ channels activation, in cardiomyocytes. They determine cardioprotection and reduce cell death and arrhythmias. The binding between adenosine agonists and its receptors induces cardioprotection, through the stimulation of NO and cysteinyl leukotrienes release [146]. Moreover, relaxin, a pleiotropic peptide hormone, acts against microvascular leakage and obstruction. Relaxin protects endothelium, regardless of NO. It promotes local inflammation suppression and protection against endothelial leakage, through VE-cadherin preservation [152]. Other pharmacological agents, in particular, resveratrol, L-Arginine, low molecular weight heparins, sodium nitroprusside, methylene blue, N-hydroxy-nor-L-arginine (NOHA), L-type calcium channels blockers act on several pathways involved in the cardioprotection, such as intracellular calcium homeostasis, NO production and release, strengthening of antioxidants systems, cellular and mitochondrial ion channels modulation [146].

### 3.2. The Functional Point of View of Coronary Microvascular Dysfunction Pathophysiology

IHD pathophysiology is complicated and multifaceted and it is not only attributable to the simplistic obstructive CAD. Atherosclerosis is just one of multiple components playing in a complex pathophysiological process that includes inflammation, thrombosis, CMD and impaired angiogenesis, underlying the “elusive link” between CAD and IHD [135,153]. Positron emission tomography (PET) scan confirms the clinical findings, according to which a patient with critical CAD may not always express myocardial ischemia and a patient with myocardial ischemia does not always show a critical CAD. In fact, comparing myocardial flow reserve by PET, patients with 100% coronary occlusion may present normal myocardial perfusion while no coronary occlusions may associate with abnormal myocardial flow [154]. Moreover, cardiac death rate at 12 months may be similar between patients with CAD and patients without obstructive CAD. Non-cardiac death rate may be higher at 30 days and 1 year, for patients with non-coronary obstructive stenosis [155,156]. Those patients show myocardial ischemia and coronary vascular dysregulation, due to endothelial and/or non-endothelial dependent microvascular dysfunction. In fact, endothelial dysfunction limits the protective effect of endogenous anti-inflammatory systems within endothelial cells, causing anomalous smooth muscle tone that impacts on myocardial perfusion [157].

Microvascular dysfunction worsens in hyperglycemic and glucose intolerance conditions because they predispose to microvascular inflammation and endothelial dysfunction [158]. Beyond macrovascular complications, diabetes mellitus plays a pivotal role in CMD development. Taken together, these conditions contribute to the development of diabetic cardiomyopathy, which is closely associated with IHD and heart failure. Diabetes mellitus promotes CMD determining an imbalance between myocardial metabolic demand and coronary blood flow [159,160,161]. Diabetic induced endothelial dysfunction may be related with several aspects associated with diabetic cardiomyopathy, such as autonomic dysfunction, neuroendocrine dysregulation, atherosclerotic lesions progression and complications, and microvascular dysfunction. Several aspects contribute to endothelial dysfunction and CMD in diabetes mellitus. In this regard, hyperglycemia is associated with massive AGEs and ROS production. They impair antioxidant systems and reduce NO bioavailability (Figure 1). Moreover, the endothelium-dependent vasodilation is compromised because of 20-hydroxyeicosatetraenoic acid (20-HETE) increased production, which has a vasoconstrictor effect. Hyperglycemia enhances vascular permeability, through the activation of the diacylglycerol (DAG)-PKC pathway and intracellular junctions weakening. Coronary ion channels physiological activation is associated with coronary vasodilation, but diabetes determines coronary vasomotor tone impairment due to coronary ion channels imbalance, such as K_ATP_ and transient receptor potential vanilloid type 1 (TRPV1) [159,160,161,162,163]. In patients with diabetes mellitus hyperinsulinemia and insulin resistance are often found. They have a pathogenetic role. In fact, they suppress NO production and release, promote endothelin-1 production and mitogen-activated protein kinase (MAPK) pathways stimulation. They are also associated with the increase in cytokines and free fatty acids production and PI3K/Akt pathway suppression. This latter pathway is involved in vasomotor tone regulation, under physiological conditions [159,160,161,162,163]. Several molecules used in diabetes mellitus treatment may have a beneficial impact on cardiovascular complications. Glucagon like peptide 1-receptor agonists (GLP-1) and sodium glucose co-transporter 2 inhibitors (SGLT2) may improve cardiovascular outcome, in patients with diabetes mellitus and cardiovascular disease. In particular, GLP-1 agonists may improve the endothelial function, through the stimulation of eNOS activity, the suppression of vascular adhesion molecule and plasminogen activator inhibitor type 1 expression. Moreover, they may have beneficial effects on atherosclerosis progression, promoting cardioprotection. SGLT2 inhibitors also have a cardiovascular impact in patients with diabetes mellitus, in particular in heart failure hospitalization reduction. Beyond the effect on diuresis, natriuresis, glucose urinary excretion and heart failure fluid overload, SGLT2 inhibitors have a beneficial effect on myocardial metabolism [159,160,161,162,163].

Aging is an important cause of CMD causing endothelial dysfunction, vasomotor tone imbalance and coronary flow reserve reduction [15,16]. Aging affects VSMCs and endothelial cells integrity and function. From the molecular point of view, the increased production of ROS and reactive nitrogen species (RNS) and their reduced degradation represent an important CMD pathogenetic mechanism in aged patients. ROS and RNS activate the PI3K/Ras/Akt/MAPK pathway, reducing eNOS activity. Moreover, they inactivate NO and many antioxidants systems, such as superoxide dismutase and catalase. Also endothelial phosphodiesterase type 5 (PDE-5) levels increase, reducing NO bioavailability, through cyclic guanosine monophosphate (GMPc) degradation. Moreover, aging reduces bradykinin-related vasodilation and the endothelium-dependent hyperpolarization [15,16]. Due to these molecular mechanisms, aging is associated with microvascular rarefaction, impaired angiogenesis, reduced gradient of intramyocardial perfusion pressure, increased deposition of cardiac extracellular matrix and left ventricular hypertrophy. These aspects contribute to the mismatch of the cross talk between cardiomyocytes and coronary circulation, a condition predisposing to IHD and heart failure [15,16,160,161].

Endothelial dysfunction seems to have a central role in vasospastic angina. Microvascular angina and vasospastic angina are typically associated with microvascular dysfunction in patients with ischemia with non-obstructive coronary artery disease (INOCA) [164,165]. INOCA is a relevant problem worldwide and it affects a considerable part of patients who do not show obstructive CAD at coronary angiography. Patients with INOCA show several atypical symptoms, which often remain misdiagnosed [164]. These patients showed a reduced response to acetylcholine, a marker of endothelial dysfunction and enhanced vasoconstrictor response to U46619, which is a thromboxane and endothelin-1 agonist. Easier and enhanced response to vasoconstrictor agents are due to abundance of thromboxane, endothelin-1 and ROS compared to endothelial derived hyperpolarizing factors, NO and prostacyclin. This imbalance seems to represent the molecular mechanism, which is common between microvascular and vasospastic angina, in patients with INOCA [164,165] (Figure 1). The myocardial infarction with no obstructive coronary artery disease (MINOCA) and INOCA syndromes are associated with several CVD, even stroke and peripheral arterial disease. Moreover, patients with these syndromes seem to frequently develop heart failure with preserved ejection fraction [166].

However, CAD and CMD are often coexisting pathophysiological mechanisms in IHD. Endothelial dysfunction is closely associated with the vasoconstrictor response of epicardial vessels in response to intracoronary acetylcholine infusion. The impaired endothelial function may be an early alteration in IHD and the atherosclerosis process [121]. Dysregulation of the cardiovascular autonomic system occurs often in IHD and other CVD. In healthy conditions, at rest, neural control has an irrelevant role and the regulation of coronary blood flow depends on other regulation mechanisms. However, during physical stress and pathological conditions, such as endothelial dysfunction, it assumes a central role in the regulation of coronary blood flow. In particular, coronary microcirculation mainly expresses β_2_ and α_2_ receptors, the stimulation of which is associated with coronary vasodilation. However, except α_2_ receptors, α adrenergic stimulation is associated with vasoconstrictor response, which is enhanced in CMD condition and myocardial ischemia [121,167,168]. The pathophysiological role of neural control is emphasized by the effect of α blockers in contrasting vasoconstriction associated with microvascular dysfunction. The activation of the muscarinic M_3_ receptor may associate with NO coronary induced vasodilation, pointing out a possible role of the parasympathetic system in coronary blood flow regulation [121].

Recent evidence focuses on coronary microvasculature and inflammation involvement, in the heart damage, induced by severe acute respiratory syndrome coronavirus 2 (SARS-CoV2), the causal agent of Coronavirus Disease 19 (COVID-19) [169,170,171,172,173,174,175,176,177,178]. The SARS-CoV-2 mortality rate seems higher in patients affected by CVD and hypertension in particular. Several COVID-19 infection cases may cause a “cardiovascular syndrome”, in which the clinical expression may be cardiomyopathy, myocarditis, ventricular arrhythmias, cardiac ischemic injury and myocardial ischemia with and without obstructive CAD [169,170,171,172,173,174,175,176,177,178]. Myocardial damage may be sustained by several conditions, in COVID-19. In this regard, myocarditis is an important condition of myocardial injury. The cardiomyocytes damage and molecular mimicry, together with the cytokines storm determine an autoimmune myocarditis. Its clinical manifestations appear two weeks later than the initial COVID-19 symptoms [169,170]. The cytokines storm and the pro-thrombotic state seen in COVID-19 contribute to atherosclerotic plaque destabilization and type I myocardial infarction. However, type II myocardial infarction is the main expression of IHD, in viral diseases [169,170]. Some reports described patients affected by acute myocardial injury with increase of cardiac troponin, ST-segment depression or elevation and absence of angiographic CAD. This early data suggest that the dominant cause of myocardial injury occurs in the absence of obstructive CAD for this group of patients [169,170,171,172]. Microvascular dysfunction and, in particular, endothelial dysfunction are pivotal mechanisms involved in the pathogenesis of systemic damage, induced by SARS-CoV-2. In particular, it is involved in the vascular inflammation related to acute respiratory distress syndrome (ARDS), but also in cardiovascular manifestations associated with COVID-19. Among the mechanisms, which could enhance this damage, the hyperinflammation and pro-coagulant state may be a possible explanation of microvascular dysfunction and small vessel thrombosis [169,170,171,172,173,174,175,176,177,178] (Figure 1). Endothelial dysfunction is the main mechanism leading to microthrombi formation and COVID-19-induced coagulopathy [169,170,171,172,173,174,175,176,177,178]. In this case, during the infection, an increased production of thromboxane, a reduced production of prostacyclin and an enhanced expression of von Willebrand factor have been observed. Moreover, the endothelial cells–leukocytes–platelet aggregates reduce microvascular perfusion, contributing to ischemic damage. Several authors detected SARS-CoV-2 in endothelial cells in the heart, lung, kidney, liver, brain and skin, where it may cause endotheliitis and vascular damage [169,170,171,172,173,174,175,176,177,178]. The cytokine storm, mainly characterized by interleukin-1 (IL-1) and IL-6, directly struck the endothelium, driving the pro-inflammatory gene expression in endothelial cells. The following integrins and chemokines exposure determines the amplification of inflammation cascade and the massive leukocyte recruitment [169,170,171,172,173,174,175,176,177,178]. The endothelial dysfunction in COVID-19 may also associate with the inflammation antibody-dependent enhancement (ADE), in which the virus uptake by macrophages, determined by the circulating non-neutralizing antibodies, enhances the inflammation cascade and endothelial cells activation [169,170,171,172,173,174,175,176,177,178]. Endotheliitis is associated with several consequences, such as increase of vascular permeability, thrombosis, vascular rarefaction and angiogenesis. In this case, although some differences, several cases of a Kawasaki disease-like syndrome in children affected by COVID-19 have been reported [179,180]. Moreover, dyslipidemia, diabetes mellitus and arterial hypertension, often seen in COVID-19 patients, determine the involvement of pericytes, which contributes to the pro-thrombotic state [169,170,171,172,173,174,175,176,177,178]. However, a cytokine storm related to hyperinflammation syndrome promotes the onset and following precipitation of myocardial injury [181,182]. A growing interest is focused on the angiotensin 2 converting enzyme (ACE 2), which is the SARS-CoV-2 receptor. It creates important connections between the virus replication pathway and the cardiovascular system. In this regard, all cardiovascular conditions share an imbalance of the renin–angiotensin–aldosterone system (RAAS), in which ACE 2 plays a central role [183]. SARS-CoV-2 infection is triggered by binding of the virus spike protein to ACE2, which is highly expressed in the heart, lung and kidney. SARS-CoV-2 mainly invades alveolar epithelial cells, causing respiratory symptoms and atypical pneumonia [183,184,185]. ACE 2 is involved also in myocardial recovery, in response to injury associated with several pathological conditions, such as heart failure [183,186,187]. Conversely, the “angiotensinic storm”, characterized by the coexistence of all the pathophysiological effects of Angiotensin II, may occur during the earlier phases of COVID-19 infection assuming a pivotal role in the COVID-19 myocardial damage and coronary microcirculation involvement. In this field, there are many studies on the ACE-2 role and its relationship with COVID-19 infection, in the microvascular dysfunction determination [182]. In this case, heart pericytes have a high expression of ACE-2, leading to the hypotheses that the virus mediated myocardial injury could associate with microvascular impairment [183,186,187,188]. The role of ACE-2 has been analyzed also in relation to its virus mediated down-regulation, which may contribute to the COVID-19 related inflammatory reaction [183,186,187,188]. Several hypotheses about treatment against cardiovascular involvement in COVID-19 are now ongoing. Alpha-1 adrenergic receptor antagonist assumption is associated with reduced mortality and necessity of ventilation in patients with ARDS. Alpha blockade contrasts the cytokines storm and the ARDS, through the reduction of the catecholamines’ effects [169,170,189]. According to the beneficial and protective effects of statins on endothelial function, they may contrast vascular damage, in COVID-19 patients. Moreover, protective effects on endothelial function and against pro-inflammatory and pro-thrombotic state in influenza have been described [169,170,190,191]. Angiotensin receptor blockers, monoclonal antibodies, which bind ACE2, synthetic ACE2 and Angiotensin 1–7 peptides [169,170,192,193], were proposed to contrast the RAAS imbalance, while dexamethasone to contrast the cytokine storm [169,170,194].

Microvascular involvement has been observed in other pathological conditions, such as acute stress-induced cardiomyopathy. Acute stress-induced cardiomyopathy, also known as Takotsubo cardiomyopathy, defines a syndrome in which the presentation may mimic ACS. Patients with Takotsubo cardiomyopathy have experienced intense psychological and/or physical stress, during the period prior to the onset of the symptoms. Acute stress-induced cardiomyopathy may be characterized by severe, but reversible, left ventricular function depression with several patterns of impaired myocardial contractility, according to myocardial adrenoceptors distribution and the disease’s anatomic substrate [195]. The absence of epicardial obstructive CAD is a typical feature of Takotsubo cardiomyopathy, but CAD presence does not exclude this syndrome. From the pathophysiological point of view, several mechanisms are involved in the myocardial damage due to Takotsubo cardiomyopathy. Sub-occlusive epicardial vessel’s spasm, as well as spontaneous coronary dissection have been observed [195,196]. However, in Takotsubo cardiomyopathy, CMD has been described as a central pathophysiological mechanism [195,197], as demonstrated by the detection of an increased microvascular resistance index (IMR), which has been also identified during the acute phase of myocardial infarction [195,198]. Although Takotsubo cardiomyopathy is excluded from the definition of MINOCA diagnosis, STEMI and NSTEMI may promote the onset of Takotsubo cardiomyopathy [199,200]. According to the role of CMD in the Takotsubo cardiomyopathy pathophysiology, endothelial dysfunction may represent the mediator between myocardial injury and stressors. Endothelial dysfunction is involved in the microvascular and epicardial arteries spasm, which leads to transient ischemia and myocardial stunning. Post-menopausal women are particularly struck by Takotsubo cardiomyopathy. In this population, endothelial dysfunction may be enhanced by estrogen deficiency, with a resulting imbalance in vasomotor tone regulation. Endothelial dysfunction is also sustained by the presence of cardiovascular risk factors, such as diabetes, arterial hypertension and dyslipidemia, which often affect Takotsubo cardiomyopathy patients. However, in Takotsubo cardiomyopathy, the main pathogenetic mechanism is the sympathetic activation, which leads to circulating catecholamines and stress-associated neuropeptides increase. Moreover, an increase of norepinephrine release, by cardiac nerve pre-synaptic ends, as well as the reduction of its re-uptake, have been identified [201,202]. These mechanisms determine the continuous and massive cardiomyocyte exposition to catecholamines. Catecholamines storm causes an imbalance of cardiomyocyte metabolic state, through a mismatch between oxygen demand and supply ratio, an increase in ROS production, the depletion of antioxidant systems and the intracellular calcium overload. The latter condition causes the contraction band necrosis, a typical histopathological finding of diseases characterized by catecholamine excess. Moreover, catecholamines storm hits coronary microcirculation, the main site of coronary resistance regulation. At microcirculation, α1 and α2 adrenoceptors mediate catecholamine induced vasoconstriction, leading to coronary blood flow regulation impairment and, therefore, myocardial ischemia [195,201]. Also, inflammation plays a role in Takotsubo cardiomyopathy. The presence of a macrophage infiltration in the myocardium driven by CXCL1 and interleukin-8 (IL-8) has been demonstrated. In particular, a decrease in CD14^+^CD16^++^ monocytes sub-population involved in the tissue repair has been observed, as well as for the intermediate monocytes sub-population CD14^++^CD16^+^. Conversely, an increase of the classical sub-population CD14^++^ CD16^−^ has been demonstrated. Moreover, the intermediate sub-population CD14^++^CD16^+^ showed a slow turnover, after the acute phase of the disease. This appears in contrast with monocytes activation, which occurs during myocardial infarction. In the Takotsubo cardiomyopathy, the slow evolution of the inflammatory response determines a typical low grade, chronic myocardial inflammation [203].

### 3.3. Microvascular–Myocardial Interaction in the Regulation of Coronary Blood Flow

In normal conditions, the heart spends much more oxygen (10 mL O_2_/min/100 at rest) compared to other organs, because of its continuous work. Myocardial oxygen extraction amounts up to ∼75% in the left ventricle, thus oxygen delivery can increase only by increasing coronary blood flow. To maintain an adequate oxygen and energetic substrate amount to every cardiomyocyte, coronary vessels show a highly developed vascular network with highly organized flow regulatory mechanisms [204,205,206,207,208,209,210]. Along the coronary tree, the distal vessels, arterioles and capillaries, with a diameter of 50–100 μm, constitute approximately 40–50% of total coronary resistance. Small arteries, ranging from nearly 100 to 200–250 μm, offer a small fraction of resistance (15–20%). Instead, large epicardial arteries provide just a minuscule fraction of resistance to coronary blood flow, as they are capacitance vessels. It is not just a diameter issue. Different studies demonstrate that the heterogeneous heart perfusion is strictly related to the structural terminal vasculature heterogeneity. This implies that also the metabolism heterogeneity may result from the tissue function adaptation to oxygen availability [211,212,213,214]. It is unknown what microvascular heterogeneity degree contributes to pathological conditions and how microvascular heterogeneity is influenced by changes in upstream coronary function [213].

**Figure 2:**
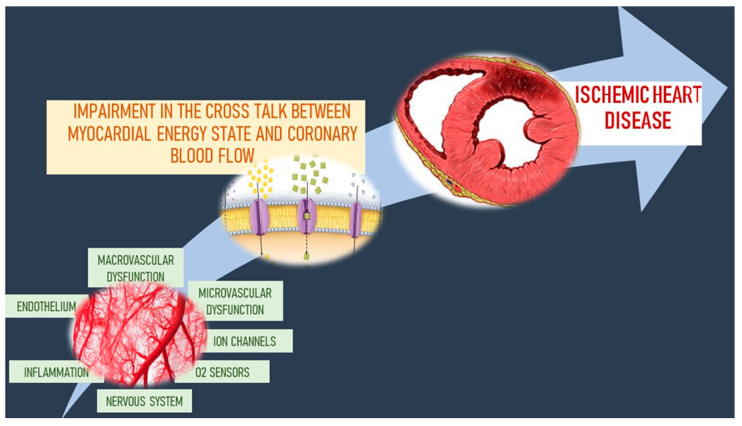
Mechanisms involved in ischemic heart disease pathophysiology and coronary ion channels role. Ischemic heart disease hides a multifaceted and complex pathophysiological paradigm. Several pathways are involved in the ischemic heart disease pathophysiology (i.e., micro and macrovascular dysfunction, atherosclerotic plaque rupture, inflammation, endothelium dependent and independent dysfunction, ion channels and nervous system impairment). In particular, coronary ion channels, represented in the central part of the figure, are the final effectors of coronary blood flow regulation mechanisms, playing a pivotal role in the coupling between myocardial metabolism and coronary circulation. Their activity dysregulation may occur during coronary microvascular dysfunction and other pathological conditions. It causes the impairment in the cross talk between myocardial energy state and coronary blood flow, leading to ischemic heart disease.

In normal coronary microcirculation, some regulators act at specific functional sites modulating blood-flow according to myocardial work. They are the endothelium, the nervous system, the auto-regulation mechanism and the myocardial metabolism. Endothelial dysfunction plays an important role in the IHD development through the vascular tone, platelet activity, leukocyte adhesion and coagulation regulation. It is a predictor of IHD in patients with and without obstructive CAD. Impaired endothelium-dependent dilation, which has traditionally been considered a precursor of atherosclerosis, is now accepted as an independent predictor of IHD in women with no or minimal epicardial stenosis and suspected ischemia. Results from the Women’s Ischemia Syndrome Evaluation (WISE) suggest that nearly 30% of women presenting with symptoms/signs of IHD in the absence of epicardial coronary disease have coronary microvascular dysfunction [214,215,216,217,218]. It is necessary to consider multifactorial aspects of IHD, in particular focusing on the microvasculature, which can be a leading cause of myocardial ischemia. Vascular oxygen sensing has intrigued and puzzled physiologists for more than 50 years. Despite some controversies, significant progress has been made in identifying candidate sensor, mediator, and effector systems in different vascular beds. There is a general agreement that oxygen sensors typically reside in the cardiac myocytes and, in minor part, in the VSMCs. They respond to oxygen tension changes, inducing redox signals/mediators that, in turn, regulate critical cellular effector systems. Under hypoxia, endothelium releases vasoactive substances that modulate vascular tone. However, the basis for the different oxygen sensing systems within the vascular system remains not fully understood. When completely understood, hoping in the near future, the basis for this critical difference will shed more light on the molecular basis of vascular oxygen sensing [219,220,221]. Hydrogen peroxide (H_2_O_2_), a product of mitochondrial electron transfer, is produced during mitochondrial electron flux and oxygen consumption. It is produced during myocardial work and it is a metabolic dilator. The H_2_O_2_ production results from an “error” in electron transfer: electrons leak from the mitochondrial complexes; these leaked electrons reduce oxygen to form the superoxide anion (O^2^¯). In mitochondria, superoxide anions are quickly mutated in H_2_O_2_. Catabolism of H_2_O_2_ by catalase and the blockade of the ionic mechanism, by which H_2_O_2_ produces dilation, attenuate coronary metabolic dilation. In the coronary circulation, H_2_O_2_-induced dilation appears to be mediated by thiol dependent voltage-dependent K^+^ channel (K_v_) channel activation [222,223,224] (Figure 1). Another important aspect to consider is the “receptors” that react with H_2_O_2_, as well as other vasoactive metabolites. Ion channels, mainly expressed in the microcirculation, are the end effectors of all the coronary blood flow regulation mechanisms. They have an important role in the regulation of coronary resistances. For this reason, they are crucial for the adaptation of coronary blood flow to cardiomyocytes’ metabolic demand. A change in their activity, such as their loss or gain of function, may be genetically determined, but also associated with cardiovascular risk factors exposition. It may occur in the pathophysiology of coronary microvascular dysfunction and myocardial ischemia [12,134,160,161,225] (Figure 2). In fact, a way to decipher critical elements of the pathway for coronary metabolic dilation consists in determining genetic associations in genes encoding for coronary blood flow regulators (i.e., ion channels, nitric oxide synthase, and sarco/endoplasmic reticulum Ca^2+^-ATPase) with the susceptibility for microvascular dysfunction and IHD [160,161,224,225]. Recently it has been reported that in vascular smooth muscle, K_v_1.5 channels play a critical role in coupling myocardial blood flow to cardiac metabolism [224]. Moreover, the prevalence of SNPs in genes encoding coronary ion channels has been studied among patients with CAD or microvascular dysfunction and those with both anatomically and functionally normal coronary arteries [226]. In this case, associations among SNPs and IHD, in terms of CAD and microvascular dysfunction, are possible. In fact, a genotype for a K_ATP_ channels subunit (i.e., rs5215_GG for Kir6.2 subunit) appears to be an independent protective factor against the development of IHD [226], illustrating a potentially important implication of genetic polymorphisms in the susceptibility to IHD. However, the precise manners through which specific genetic polymorphisms affect ion channel function and expression have to be clarified. Absence of coronary ion channels may compromise association between metabolism and flow, resulting in cardiac pump dysfunction and tissue hypoxia, when metabolic demand is increased, during a norepinephrine stress test. Physiologic coronary blood flow regulation depends on several ion channels, not only voltage-gated potassium (K_v_) channels, but also ATP-sensitive potassium (K_ATP_) channels, voltage-gated sodium (Na_v_) channels, to name just a few of them (Figure 1). In this regard, several drugs such as Nicorandil and Levosimendan, which may be used in angina and heart failure treatments respectively, determine coronary vasodilation, inducing K_ATP_ opening [227]. Ion channels play an important role in calcium regulation and concentration of both coronary smooth muscle and endothelial cells. They respectively modulate the contractile tone degree, in vascular smooth muscle, and the amount of NO, produced by the endothelium. In this context, they contribute to the coronary arterioles response to myocardial metabolic demands [12,134,160,161]. For this reason, they are involved in the IHD pathophysiology and other conditions in which IHD may be associated, such as heart failure, the pathophysiology of which is also multifaceted and complex [228,229,230,231,232,233,234].

## 4. Conclusions

IHD occurs as the result of multiple altered regulating vascular pathways that include severe atherosclerosis just in some cases. IHD pathophysiology is complex and multifaceted and a panoramic overview about its current paradigms is shown in Figure 1. A large percentage of patients with IHD have minimal or no epicardial coronary vascular disease. In fact, the atherosclerotic point of view has been revised by a number of trials and studies. They suggest that microvascular disease plays an important role in the etiology of IHD, by regulating blood flow and oxygen and energetic substrates delivery, in the microcirculation–myocardium interaction. Our current understanding about this process involves the feedforward production of H_2_O_2_ that cause vasodilation during increased cardiac work. However, there are other key metabolites over H_2_O_2_, and many proteins, such as K_v_1.5 channels, Na_v_ channels, K_ATP_ channels, that represent the final effectors of coronary blood flow regulation mechanisms. They contribute in different ways to the regulation of intracellular calcium, the degree of contractile tone, and ultimately, coronary blood flow. In our viewpoint, myocardial ischemia is directly dependent on an impairment of the cross talk between myocardial energy state and coronary blood flow. Coronary macrovascular and microvascular disease may represent just a portion of the multifaceted pathophysiology of myocardial ischemia. More bodies of evidence are needed in order to provide a deeper understanding of IHD underlying mechanisms, almost occurring in the most distal and microscopic segments of the coronary tree.
